# Toward a New Conceptual Framework for Digital Mental Health Technologies: Scoping Review

**DOI:** 10.2196/63484

**Published:** 2025-02-19

**Authors:** Gareth Hopkin, Holly Coole, Francesca Edelmann, Lynda Ayiku, Richard Branson, Paul Campbell, Sophie Cooper, Mark Salmon

**Affiliations:** 1 Science Evidence and Analytics Directorate, National Institute for Health and Care Excellence (NICE), Manchester, United Kingdom; 2 Software Team, Healthcare Quality and Access Group, Medicines and Healthcare products Regulatory Agency (MHRA), London, United Kingdom

**Keywords:** digital mental health, digital health, mental health, eHealth, categorization, conceptual, framework, regulation, synthesis, review methods, review methodology, systematic

## Abstract

**Background:**

Digital mental health technologies (DMHTs) are becoming more widely available and are seen as having the potential to improve the quality of mental health care. However, conversations around the potential impact of DMHTs can be impacted by a lack of focus on the types of technologies that are available. Several frameworks that could apply to DMHTs are available, but they have not been developed with comprehensive methods and have limitations.

**Objective:**

To address limitations with current frameworks, we aimed to identify existing literature on the categorization of DMHTs, to explore challenges with categorizing DMHTs for specific purposes, and to develop a new conceptual framework.

**Methods:**

We used an iterative approach to develop the framework. First, we completed a rapid review of the literature to identify studies that provided domains that could be used to categorize DMHTs. Second, findings from this review and associated issues were discussed by an expert working group, including professionals from a wide range of relevant settings. Third, we synthesized findings to develop a new conceptual framework.

**Results:**

The rapid review identified 3603 unique results, and hand searching identified another 3 potentially relevant papers. Of these, 24 papers were eligible for inclusion, which provided 10 domains to categorize DMHTs. The expert working group proposed a broad framework and based on the findings of the review and group discussions, we developed a new conceptual framework with 8 domains that represent important characteristics of DMHTs. These 8 domains are population, setting, platform or system, purpose, type of approach, human interaction, human responsiveness, and functionality.

**Conclusions:**

This conceptual framework provides a structure for various stakeholders to define the key characteristics of DMHTs. It has been developed with more comprehensive methods than previous attempts with similar aims. The framework can facilitate communication within the field and could undergo further iteration to ensure it is appropriate for specific purposes.

## Introduction

In recent years, the availability of digital mental health technologies (DMHTs) has rapidly expanded [1]. These technologies are seen as having a high potential for improving mental health care and are the focus of initiatives to improve health care across the world [2]. However, conversations around the potential impact of DMHTs can be impacted by a lack of focus on the types of technologies that are available. This can hinder discussions about the opportunities and challenges associated with specific characteristics of DMHT [3]. Clearer frameworks outlining the key characteristics of DMHTs could aid these discussions and have benefits for a range of purposes.

A key step for developers preparing to meet regulatory requirements is developing an intended purpose statement [4,5]. Clarity around the key characteristics of DMHTs within a defined framework could help developers to ensure that these statements provide sufficient detail. It could also help developers and other stakeholders to interpret whether technologies qualify as software as a medical device and how it should be classified. In addition, there may be benefits for the postmarket surveillance capability of regulators allowing safety events for certain types of DMHTs to be identified more effectively [6]. In some jurisdictions, there may also be benefits in providing clarity around where regulatory discretion applies [7].

Clearer frameworks for categorization could also have benefits within research and evaluation, including health technology assessment (HTA). To conduct systematic reviews and economic evaluations of DMHTs, one must understand whether technologies are similar enough to be compared. This is important to help determine if assumptions of similarity can be met within meta-analysis or to inform subgroup analyses [8]. If the similarity of DMHTs and their mechanism of action are not considered and they are not appropriately grouped together, then meta-analyses are likely to have unacceptably high levels of heterogeneity, even using random-effects methods. This may lead researchers to be unable to identify DMHTs that are most likely to be beneficial and to make helpful recommendations on their use. Within economic analysis, it is also important to assess whether estimates of effectiveness are likely to be generalizable across DMHTs and whether interventions are likely to have similar implications for benefits and costs that are accrued over time [9]. A better definition of types of DMHTs and key characteristics could also ensure that recommendations from HTA agencies (eg, the National Institute for Health and Care Excellence [NICE] in England or the Canada’s Drug Agency in Canada) are targeted at a suitable group of technologies.

Several categorization or classification frameworks for digital health technologies are available within regulation and HTA. These are designed to be broad and appropriate for a range of digital technologies across health conditions and have broad international applicability [10,11]. These frameworks categorize technologies according to the severity of the conditions and their role in clinical management. However, there is uncertainty around how to place DMHTs within these categories. For example, the NICE evidence standards framework for digital health technologies categorizes technologies with direct health outcomes into those that inform treatment, drive treatment, treat a specific condition, or diagnose a specific condition [11]. These are broad categories that may suit the goal of defining appropriate evidence. However, these categories are not able to distinguish between types of DMHT at a more granular level, such as between a cognitive behavioral therapy (CBT) app with high levels of personalization that relies on artificial intelligence (AI) to tailor treatment and a CBT app that provides a fixed digitized version of written materials. Within regulation, nomenclature coding systems are also available to categorize technologies [12,13], and specific codes for DMHTs are needed. However, at present, these codes to categorize DMHTs vary in the level of detail they provide and may include a large number of technologies with differing characteristics. For example, an existing code for psychological assessment software will encompass DMHTs that have a wide range of intended purposes and different potential benefits and risks.

Within published literature, there have been limited attempts to provide frameworks to categorize DMHTs. These existing taxonomies or frameworks focus on specific clinical areas, such as use within primary care [14], or focus on issues important for research, such as distinctions between active interventions and interventions used as comparators within control arms of randomized trials [15]. There are methodological limitations within these existing studies, and they do not identify studies that were available at the time of publication and were identified during scoping for this review. In addition, we are not aware of any framework for categorizing DMHTs that has been supported by both a review of existing work and consensus from experts. Due to the lack of rigorous work in this area, there have been calls from researchers and other stakeholders to develop improved frameworks for DMHTs to help address issues across research, evaluation, regulation, and HTA [15,16].

To address this gap, we aimed to identify existing literature on the categorization of DMHTs, to explore challenges with categorizing DMHTs for specific purposes, and to develop a new framework. This work is focused on DMHTs for several reasons. First, mental health conditions can present differently and require different approaches to other conditions both due to their spectrum-based nature spanning general well-being to more severe presentation and the highly individualized nature of risk. Second, DMHTs themselves can take an active role in managing people’s symptoms or conditions with partial or no support from other professionals. This is less likely to occur in physical health conditions. Similarly, there are also digital health technologies that may play a substantial role in physical health but are not likely to be adopted for mental health [17]. There will inevitably be overlaps with how digital health technologies for other conditions could be categorized. However, our focus allows greater consideration and contextualization of issues relating to mental health and the nuances of delivering mental health care and aims to provide a more tailored framework that focuses on the types of digital health technologies available for mental health.

## Methods

### Overview

We used an iterative approach with several steps. In step 1, we completed a rapid review to identify previous literature that has attempted to categorize DMHTs. In step 2, we discussed findings from the rapid review and broader issues with developing a framework with an expert working group. Finally, in step 3, we considered the perspectives of the expert working group and developed a framework that could inform future work in this area.

### Step 1: Identifying Previous Work on Categorizing DMHTs

A rapid review was developed in line with best practice recommendations [18], and preferred reporting items according to PRISMA (Preferred Reporting Items for Systematic Reviews and Meta-Analyses) 2020 are provided where applicable [19]. A completed PRISMA checklist is provided in Checklist 1.

#### Search Strategy

We searched the following electronic bibliographic databases of published studies: Embase (Ovid), MEDLINE (Ovid), PsycINFO (Ovid), Health Management Information Consortium, HMIC (Ovid), and Epistemonikos between August 29 and September 2, 2023. We also checked the reference lists of retrieved papers for other relevant materials and searched Google to identify gray literature such as reports and policy documents. An information specialist conducted the search with terms based on MeSH and text words from key papers that were identified during scoping. The review followed an unpublished protocol and was not registered.

The search was divided into three sets of terms targeting the expected types of studies that (1) aimed to develop a taxonomy (eg, terms including taxonomy, typology, categorization, and group), (2) provided a narrative review of the field of digital mental health (eg, terms including field, future, advance, emerging, and state), and (3) completed a systematic review grouped by type of technology (eg, terms including review, synthesis, and analysis). More details on the search strategy are available in Multimedia Appendix 1.

#### Eligibility Criteria

Studies were considered eligible for inclusion if they provided an approach to categorizing or grouping digital technologies according to their characteristics and had a specific focus on mental health. The digital technologies of interest in this review are software that aims to have a direct or indirect impact on an individual’s health outcomes through use by the individual or a professional. We did not include digital technologies that aim to improve population-level care or improvements in efficiency. This is in line with the definition used in other frameworks [10,11]. Both qualitative and quantitative studies were also included if they met these inclusion criteria. Studies were not included if they extracted characteristics of technologies but did not provide clear groupings. We only included English language studies.

#### Study Selection and Data Extraction

One team member (LA) completed the initial screening of studies by title. A second team member then screened potentially relevant studies by title and abstract (GH). For eligible studies, data were extracted using a standardized data extraction form by 1 team member (GH) and reviewed by a second team member (FE).

Information extracted included the aim and scope of the study, type of study, and details of domains used to group technologies. Initially, domains used to group technologies were extracted according to the name provided by the authors. Domains were then reviewed for similarity and pooled if appropriate. The assessment of whether a domain was present within a study was made based on the prominence given by the authors. Domains were included where a list of types of technology was provided within a paper, where a characteristic was provided as a subheading, or where a characteristic was used to define a subgroup within an analysis. For example, one paper describing a framework for mental health in primary care provides a list of domains that can be used to categorize DMHTs [14]. Similarly, in a systematic review on interventions for university students, DMHTs are divided into domains in the narrative synthesis of results [20].

### Step 2: Discussion Within an Expert Working Group

#### Group Membership

The findings of the rapid review and challenges associated with categorizing DMHTs were considered by an expert working group during a web-based meeting in November 2023. This is a group that has been convened to meet and provide guidance for the duration of a Wellcome-funded research project [21].

The group was formulated to provide expertise on digital mental health from across the field. Experts were selected based on their experience of designing and researching DMHTs, using DMHTs in clinical practice, and managing the deployment of DMHTs in health services. The group comprises representatives from the Medicines and Healthcare products Regulatory Agency and NICE (including members of the project team and other experts from these organizations), representatives from NHS England, a variety of health professionals with experience of mental health conditions (eg, clinical psychology, nursing, psychiatry, and general practice), academics, representatives from health innovation networks, and lived experience advisers.

#### Content of Discussions

During the meeting, the group was asked to consider a series of questions through facilitated discussions. These questions related to (1) the value of developing a framework to categorize DMHTs, (2) the practical purposes of a framework, and (3) the drawbacks or challenges of developing a single framework for these purposes. Emerging themes from these discussions were identified and considered by the research team.

Within smaller breakout groups, members were then asked to consider (1) which of the domains identified within the review would be most important to include in a taxonomy, (2) whether additional domains that captured other characteristics of DMHTs were needed, and (3) whether descriptions of the domains reflected anticipated content and whether adaptations were needed to these descriptions. Based on this, groups were asked to define which domains should be included within a new framework for categorizing DMHTs and how they should be conceptualized. The domains proposed for inclusion and other considerations were collated to support the development of the new framework.

To support these discussions, the group were provided a summary of the rapid review methods, and results described within this paper, along with a sample of the publications included within the review.

### Step 3: Developing a New Conceptual Framework

Members of the research team synthesized findings from the rapid review and the expert working group. We considered the domains identified in the initial rapid review, additional domains or refinements suggested by the working group, and the similarities and differences between domains that the breakout groups recommended for inclusion. We also considered the general reflections of the group on the value of developing a framework and the appropriate structure. Based on these discussions, our final framework was developed.

## Results

### Step 1: Identifying Previous Work on Categorizing DMHTs

#### Search Results

**Figure 1.**
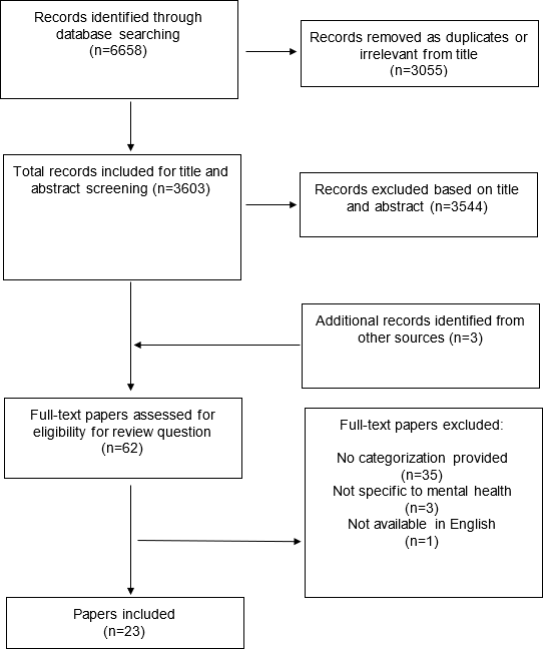
PRISMA (Preferred Reporting Items for Systematic Reviews and Meta-Analyses) diagram for rapid review.

The search returned a total of 3603 unique results from databases and other sources (Figure 1). Of these, 3544 were excluded by title and abstract not adequately reflecting the subject matter, and 59 of these full-text papers were subsequently reviewed. A further 3 studies that were not identified by the search were included after hand searching references and included in the full-text review. After review, 23 papers were considered eligible for inclusion.

#### Study Characteristics

Most studies were systematic reviews of the effectiveness or quality of technologies and grouped technologies to support analysis (8/23, 35%). The remaining studies either provided groupings of types of technologies as part of commentaries or narrative reviews of the field of digital mental health (7/23, 30%), aimed to develop their own taxonomy (5/23, 22%), or explored the implementation of DMHTs in mental health services with qualitative methods (1/23, 4%). In total, 1 (4%) study was a consensus statement on the use of DMHTs in the United States, and one further was public information for Canadian people on how to select appropriate technologies.

Most studies included DMHTs across a range of conditions and stages of the treatment pathway (15/23, 65%), but some had a more specific focus. This included anxiety and depression (2/23, 9%), comorbid mental and physical conditions (1/23, 4%), mental health during COVID-19 (1/23, 4%), youth mental health or university students (1/23, 4%), management in primary care (1/23, 4%), and ante- and postnatal mental health (1/23, 4%).

The majority of studies did not define how domains used to categorize technologies were generated (11/23, 48%), although some outlined underlying methods based on previous taxonomies (4/23, 18%) or coding characteristics of relevant technologies in systematic reviews (6/23, 26%). One study reported using workshops to develop consensus on appropriate groupings, and one was based on themes arising from qualitative interviews.

#### Domains

Ten domains were identified across the included studies in line with the method outlined earlier. These are condition, setting, platform or system, function, subfunction, professional input, type of communication, type of intervention, sophistication, and consumable resource use. Definitions for these domains are provided in Textbox 1.

Textbox 1. **Definitions of domains extracted from included studies within rapid review.**

Condition: this relates to the condition that the digital mental health technology (DMHT) is targeting. In some studies, this was narrowed to include a more specific population (eg, people with anxiety and other comorbid conditions).

Setting: this refers to the health care setting in which the DMHT is being delivered. Available studies categorized settings in different ways, including inpatient, outpatient, and aftercare services.

Platform or system: this refers to the platform or system that is used. This could be related to hardware (eg, smartphone) that is used to access the DMHT or be related to the content of the DMHT itself (eg, chatbot).

Function: this refers to the overarching aims of the DMHT and whether it provides assessment and diagnosis, treatment, monitoring and support, decision support, or other functions.

Subfunction: this relates to the function of the DMHT but provides an additional level of detail with more specific details of what the technology does.

Professional input: this refers to the extent of input from health professionals. DMHTs can be unguided with limited or no input from professionals, guided with professionals providing some input, or supervised with technologies being used during in-person contacts.

Type of communication: this refers to how a user and professional are able to communicate. Within studies, communication is usually described as synchronous and in real time (eg, through a live video feed) or asynchronous with information being transmitted and reviewed at a later time.

Type of intervention: this refers to a type of therapy or other intervention that is provided. All studies related this domain to treatment (as opposed to other functions like diagnosis or monitoring and support) and usually delineated types of therapy (eg, cognitive behavioral therapy and other approaches).

Sophistication: this refers to the extent to which a technology differs in function to nondigital versions. Within the relevant study, digitized refers to content that is converted from previously available analog resources with limited changes or additions, whereas digital refers to content that is only possible with digital technology.

Consumable resource use: this refers to whether interventions require a resource that is “used up or consumed.” For DMHTs, this refers to whether professionals are involved in delivery and are required to spend time either inducting a user or spending additional time reviewing inputs that would otherwise have been discussed in prearranged appointments. Professionals working in the field of mental health often have restricted capacity based on the availability of the workforce.

Studies included domains on professional input (11/23, 48%), type of platform or system used (10/23, 43%), target condition (6/23, 26%), function (5/23, 22%) and subfunction (2/23, 9%) of the technology, type of intervention (4/23, 17%) with each of these was focused on the type of psychological therapy, the setting (4/23, 17%), timing of communication with professionals (2/23, 9%), whether a consumable resource was used (2/23, 9%), and technical sophistication (1/23, 4%). Details on the domains included within each study are shown in Table 1.

**Table 1.** Presence of identified domains in each of the included studies from the rapid review.

	Condition	Setting	Platform or system	Function	Sub function	Professionalinput	Type of communication	Type of intervention	Sophistication	Consumable resource used
Baños et al (2022) [22]			✓							
Burger et al (2020) [23]	✓		✓	✓	✓	✓		✓		
Cross et al (2023) [24]		✓				✓				
Dülsen et al (2020) [25]		✓				✓				
Gagnon et al (2022) [14]			✓	✓	✓	✓	✓	✓		
Gega et al (2022) [15]						✓				
Gooding and Kariotis (2021) [26]			✓	✓						
Harith et al (2022) [20]		✓	✓					✓		
Lattie et al (2022) [27]						✓		✓		
Lau et al (2024) [28]	✓		✓							
Li (2023) [29]										
Lukka et al (2023) [30]				✓					✓	
Mental Health Commission of Canada (2020) [31]			✓	✓						
Mohr et al 2023 [32]						✓				
Muñoz et al 2018 [33]						✓				✓
Nuffield Council on Bioethics, 2022 [34]			✓							
Philippe et al, 2021 [35]	✓		✓				✓			
Pineda et al 2023 [36]	✓					✓				✓
Rickard et al 2022 [37]				✓						
Sasseville et al, 2023 [38]	✓		✓			✓				
Schueller et al, 2020 [39]		✓				✓				
Shatte et al 2019 [40]				✓						
Torous et al 2021 [41]	✓		✓							

### Step 2: Discussion Within an Expert Working Group

There were a series of key reflections from the expert working group. The group saw the benefits of a framework to categorize technologies and suggested that this would be useful for a wide range of health system stakeholders. A framework to categorize DMHTs could provide clarity around common characteristics of DMHTs and could also assist communication between health professionals and across multidisciplinary boundaries.

However, the group also identified a number of challenges in developing a framework for categorizing DMHTs. First, a single framework would be too generic for the specific purposes of different stakeholders. Second, there are practical challenges around categorizing DMHTs that have multiple functions and the potential for a categorization system to become unwieldy.

The group highlighted that a solution would be to develop a broad overarching framework that could then be adapted by stakeholders for specific purposes. This would provide a common language and consistent definitions for describing the characteristics of DMHTs. It could also then be refined by other stakeholders in consultation with experts in a specific field. This may involve reducing it to key domains for a specific purpose or refocusing the framework in other ways, if needed.

There was some divergence in opinion across breakout groups on which domains should be included within a framework. Some groups reduced the number of domains to those they felt were most important. Others indicated that all of the included domains were necessary but suggested merging or adapting some domains to retain information in a streamlined way. There were several suggestions on how the domains should be described and what they should include. Inclusions of domains from the rapid review and suggested adaptations are summarized in Multimedia Appendix 2.

### Step 3: Developing a New Conceptual Framework

#### Overview

**Figure 2.**
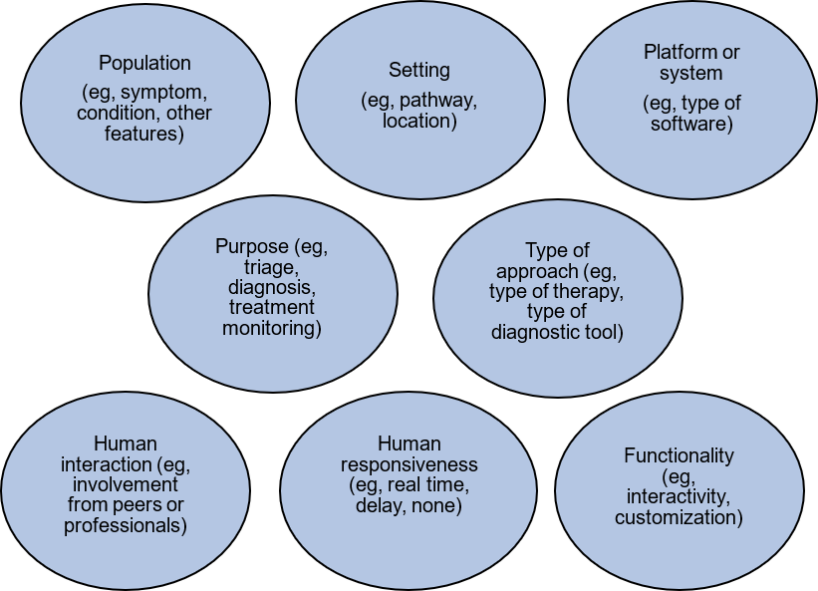
Domains included within a new conceptual framework.

Based on the findings of the literature review and reflections from the working group, we developed a conceptual framework for DMHTs. The conceptual framework includes 8 domains that represent the characteristics of DMHTs (Figure 2). They each represent important characteristics of DMHTs that are likely to influence the benefits they can deliver for individuals and the wider system and risks that will need to be mitigated during use.

The proposed conceptual framework also uses a “tagging” approach, where multiple attributes within a single domain could be attributed to a technology (eg, the population targeted by a DMHT), rather than seeking to develop an approach using “buckets” to assign a particular technology to a discrete group based on a characteristic.

#### Proposed Domains

##### Population

This refers to the population that a DMHT is intended to benefit. The population could be defined by the target condition (eg, depression, anxiety, and psychosis) or specific symptoms either on a transdiagnostic basis (eg, low mood and anhedonia) or secondary to a condition (eg, agoraphobia secondary to psychosis). The severity of the condition or symptoms that the technology is aimed for (ie, mild, moderate, and severe) could also be defined. If a DMHT targets mental health symptoms within the context of other conditions or comorbidities, it would be useful to include and identify these (eg, cancer and multiple sclerosis). There may also be other important population characteristics that could be defined (eg, age and gender).

##### Setting

This refers to where in a treatment pathway, a DMHT is delivered or deployed. This information could be defined by the stage of care (eg, primary and secondary) or the type of health provider (eg, inpatient unit). It would also be possible to provide more specific detail on placement within a clinical pathway (eg, on a waiting list after the initial presentation and during in-person therapy sessions).

##### Platform or System

This domain relates to the type of software or platform that is being used to deliver the DMHT. Information about the platform or system will be key to determine how the technology is delivered and the practicalities around its use. Some types of platform or system may have substantial variation within types (eg, apps and websites), whereas others will be more specific (eg, virtual reality). There may also be cases where specific hardware needs to be specified because DMHTs are not able to rely on off-the-shelf solutions (eg, monitoring vital signs in inpatient settings).

##### Purpose

This domain relates to the primary intended purposes or aim of the DMHT. There may be different ways to define this, but this would broadly fit into categories of prevention, diagnosis, triage, treatment, and monitoring. There may be additional purposes around supporting recovery, preventing relapse, and others that should be considered as a standalone category, and consensus on available purposes for DMHTs may be helpful. DMHTs may have multiple purposes, which could be identified and “tagged” in line with the approach used in this framework (eg, diagnosis and treatment).

##### Type of Approach

This domain would provide a more specific definition or description of the approach used within the DMHT. This domain may be most appropriate for DMHTs that provide therapy and could be designed to identify the type of therapy that is being used (eg, CBT, mindfulness, and exposure therapy), but there may be other types of DMHTs that would also benefit from identifying with more detail the nature of what is being provided. For example, DMHTs providing diagnosis could be clear whether they are using validated patient-reported outcome measures or new approaches (eg, eye tracking and motion software for attention-deficit/hyperactivity disorder).

##### Functionality

This domain relates to a DMHT’s level of interactivity, customization, and complexity of software capability. This may include providing varied or bespoke content to different users and allowing users to determine the content they view through their inputs. The minimum functionality of a DHMT would be fixed content that is uniform for all users and does not allow any interactivity. The most sophisticated functionality would be DMHTs supported by generative AI that may be able to provide unique responses to inputs that are determined by a user.

##### Human Interaction

This domain relates to whether a human (other than the patient) is involved in the delivery of the DMHT. The level of human interaction is likely to be on a spectrum between no human interaction and full human supervision within an in-person session. It may also be necessary to define who is leading the human interaction. This may be a health, social care, or education professional (eg, nurse, clinical psychologist, and mental health support worker) or may be peers.

DMHTs supported by AI may have high levels of functionality and mimic human interaction but be unsupported by professionals or peers. For this reason, human interaction is specified.

##### Human Responsiveness

This domain is related to human interaction and provides details on the extent and timeliness of a human’s ability to respond to information. DMHTs have the potential to collect a large amount of quantitative and qualitative data that could help determine changing levels of risk that require intervention from a professional. Providing details on how and when a human will respond to information is therefore important in interpreting how risk is managed.

DMHTs supported by AI may be able to interpret quantitative and qualitative data. However, as mentioned earlier, a human’s ability to respond and provide professional intervention, if needed, is an important aspect of how risk is managed. The need for health professionals to monitor and respond to information gathered by DMHTs will also have implications for adoption within health services.

## Discussion

### Principal Findings

In this study, we have proposed a new conceptual framework for categorizing DMHTs. This comprehensive framework is based on the findings of a rapid review, discussions within an expert working group, and deliberations by professionals working within the regulation and evaluation of DMHTs. This represents an advance in methods compared to previous approaches, which have attempted to develop frameworks or taxonomies within this field [14,15].

### Future Directions

The proposed framework is designed to be used by stakeholders across the health system, from the initial stages of product development and market access to product selection by health professionals and users. The 8 domains were chosen because they represent key characteristics of DMHTs. They also have an important impact on the potential benefits and risks associated with the technologies and implications for implementation within health services. Indeed, the International Medical Device Regulators Forum [42] has recently completed a consultation on considerations for risk characterization for software as a medical device, and there is a strong alignment between their suggested approach and our more focused approach for mental health.

The development of this new conceptual framework presents opportunities for future work. Further research could trial this framework with a set of DMHTs and explore whether the framework provides a useful descriptive system and can group together similar technologies. This could support consideration of whether a menu of options is needed or whether there should be flexibility in assigning attributes within domains.

There are also tangible practical applications. Within regulation, there may be opportunities for the framework to be refined to support the coding of medical devices and to assist with mapping DMHTs to other regulatory frameworks. The research team is also planning to explore whether the framework can be used to provide clearer guidance on classification groups within the NICE evidence standards framework. Researchers could also adopt the conceptual framework provided within this study to give greater transparency around methodological choices within evidence synthesis and economic evaluation.

### Strengths and Limitations

This study has several strengths and limitations that should be considered. The development of the conceptual framework was initially supported by a rapid review. These methods provide a streamlined approach that can meet the needs of faster-paced activities, but this can reduce their comprehensiveness. The rapid review approach allowed literature to be identified in short timelines, prior to the expert working group. However, we are aware that some types of literature that may provide ways to categorize DMHTs were not targeted (eg, evaluation frameworks) in order to allow manageable search methods. Given the breadth of domains identified in the rapid review and adaptations suggested by the expert group, we do not anticipate that identifying a wider range of studies would have had a large impact.

The development of the framework has been led by professionals with expertise across regulation and evaluation, including HTA, where frameworks could have practical purposes. Through the expert working group, there has also been input from a wide range of professionals across disciplines. However, the proposed framework has not been through wider consultation with experts outside of this group.

Similarly, it should be acknowledged that the authors work for the Medicines and Healthcare products Regulatory Agency, which has a UK-wide remit, and NICE, which is England’s HTA agency. In addition, members of the working group largely work within the English health care system. The proposed framework is aimed at being internationally relevant and due to the global market in digital health, it is likely that professionals across the world are thinking about the same types of technologies and addressing similar challenges. However, there may be nuances from other countries that are not acknowledged here.

To address each of these limitations, we would welcome other experts within this field across disciplines and countries to interact with and test this proposed framework. This could include an exploration of whether this framework could be applied to other health conditions or whether specific domains are needed. It could also lead to further refinement based on a wider consultation than was possible in this project.

## Supplementary material

10.2196/63484 Multimedia Appendix 1 Example search strategies from MEDLINE.

10.2196/63484 Multimedia Appendix 2 Summary of domains retained by each breakout room in the expert working group and suggested adaptations.

10.2196/63484 Checklist 1 PRISMA (Preferred Reporting Items for Systematic Reviews and Meta-Analyses) checklist and PRISMA for abstracts checklist.

## Abbreviations

AI: artificial intelligence
CBT: cognitive behavioral therapy
DMHT: digital mental health technology
HTA: health technology assessment
NICE: National Institute of Health and Care Excellence
PRISMA: Preferred Reporting Items for Systematic Reviews and Meta-Analyses
