# Supplementary material for: Toward a New Conceptual Framework for Digital Mental Health Technologies: Scoping Review
Source: JMIR Ment Health. 2025 Feb 19;12:e63484. doi: 10.2196/63484 (PMC11864090; doi:10.2196/63484)
Supplement: Multimedia Appendix 1 [file mental-v12-e63484-s001.docx]

*For taxonomies*

Database: Ovid MEDLINE(R) ALL <1946 to August 28, 2023>

Search Strategy:

--------------------------------------------------------------------------------

1 (digital* and (mental* or psych*)).ti. (1266)

2 (digital* adj3 (mental* or psych*)).ab,kf. (1096)

3 dmhi*.tw,kf. (56)

4 or/1-3 (1922)

5 exp *cataloging/ or exp *classification/ (49881)

6 framework*.tw. (405289)

7 (standard or standards).tw. (1242253)

8 (taxonom* or typolog* or type* or ontolog* or categor* or catalog* or classif* or criteri* or segment* or hierarch* or defin* or organis* or organiz* or character* or group* or theme* or schem* or map* or roadmap* or "road map*" or "road-map*").tw. (12843771)

9 Software/cl [Classification] (64)

10 or/5-9 (13585923)

11 4 and 10 (1062)

12 limit 11 to english language (1030)

13 animals/ not humans/ (5116127)

14 12 not 13 (1025)

*For systematic reviews*

Database: Ovid MEDLINE(R) ALL <1946 to August 31, 2023>

Search Strategy:

--------------------------------------------------------------------------------

1 ((digital* or ehealth* or e-health* or "electronic health*" or mhealth* or "m-health*" or "mobile health*" or online or internet* or app or apps) and (mental* or psych*)).ti. (4973)

2 ((digital* or ehealth* or e-health* or "electronic health*" or mhealth* or "m-health*" or "mobile health*" or online or internet* or app or apps) adj3 (mental* or psych*)).ab,kf. (4248)

3 (emental* or "e-mental*" or "electronic mental*").tw,kf. (653)

4 dmhi*.tw,kf. (56)

5 or/1-4 (8185)

6 ((overview$ or review or synthesis or summary or cochrane or analysis) and (reviews or meta-analyses or articles or umbrella)).ti. or "umbrella review".ab. or (meta-review or metareview).ti,ab. (8918)

7 5 and 6 (39)

8 limit 7 to english language (37)

N.B. The validated search filter by Lunny, et al. (2015)^1^ was used to retrieve overviews of systematic reviews.

^1^ Lunny C, McKenzie JE, McDonald S. Retrieval of overviews of systematic reviews in MEDLINE was improved by the development of an objectively derived and validated search strategy. J Clin Epidemiol. 2016 Jun;74:107-18. doi: 10.1016/j.jclinepi.2015.12.002. Epub 2015 Dec 23. PMID: 26723872. <https://www.sciencedirect.com/science/article/abs/pii/S0895435615005788>

*For commentaries and narrative reviews*

Database: Ovid MEDLINE(R) ALL <1946 to August 31, 2023>

Search Strategy:

--------------------------------------------------------------------------------

1 ((digital* or ehealth* or e-health* or "electronic health*" or mhealth* or "m-health*" or "mobile health*" or online or internet* or app or apps) and (mental* or psych*)).ti. (4973)

2 ((digital* or ehealth* or e-health* or "electronic health*" or mhealth* or "m-health*" or "mobile health*" or online or internet* or app or apps) adj3 (mental* or psych*)).ab,kf. (4248)

3 (emental* or "e-mental*" or "electronic mental*").tw,kf. (653)

4 dmhi*.tw,kf. (56)

5 or/1-4 (8185)

6 (current or field or horizon* or future* or "next step*" or advance* or "lesson* learn*" or potential or emerg* or "real world" or "state" or priorit* or implement* or adopt* or integrat* or incorporat* or polic* or strateg* or nation* or government* or ethic* or barrier* or factor* or challeng* or opportunit* or enabl* or facilitat* or uptak* or engag* or usage or "use" or perspective* or experience* or consensus or law* or legal* or legislat* or regulat*).ti. (4827968)

7 5 and 6 (2543)

8 limit 7 to yr="2020 -Current" (1559)

9 afghanistan/ or africa/ or africa, northern/ or africa, central/ or africa, eastern/ or "africa south of the sahara"/ or africa, southern/ or africa, western/ or albania/ or algeria/ or andorra/ or angola/ or "antigua and barbuda"/ or argentina/ or armenia/ or azerbaijan/ or bahamas/ or bahrain/ or bangladesh/ or barbados/ or belize/ or benin/ or bhutan/ or bolivia/ or borneo/ or "bosnia and herzegovina"/ or botswana/ or brazil/ or brunei/ or bulgaria/ or burkina faso/ or burundi/ or cabo verde/ or cambodia/ or cameroon/ or central african republic/ or chad/ or exp china/ or comoros/ or congo/ or cote d'ivoire/ or croatia/ or cuba/ or "democratic republic of the congo"/ or cyprus/ or djibouti/ or dominica/ or dominican republic/ or ecuador/ or egypt/ or el salvador/ or equatorial guinea/ or eritrea/ or eswatini/ or ethiopia/ or fiji/ or gabon/ or gambia/ or "georgia (republic)"/ or ghana/ or grenada/ or guatemala/ or guinea/ or guinea-bissau/ or guyana/ or haiti/ or honduras/ or independent state of samoa/ or exp india/ or indian ocean islands/ or indochina/ or indonesia/ or iran/ or iraq/ or jamaica/ or jordan/ or kazakhstan/ or kenya/ or kosovo/ or kuwait/ or kyrgyzstan/ or laos/ or lebanon/ or liechtenstein/ or lesotho/ or liberia/ or libya/ or madagascar/ or malaysia/ or malawi/ or mali/ or malta/ or mauritania/ or mauritius/ or mekong valley/ or melanesia/ or micronesia/ or monaco/ or mongolia/ or montenegro/ or morocco/ or mozambique/ or myanmar/ or namibia/ or nepal/ or nicaragua/ or niger/ or nigeria/ or oman/ or pakistan/ or palau/ or exp panama/ or papua new guinea/ or paraguay/ or peru/ or philippines/ or qatar/ or "republic of belarus"/ or "republic of north macedonia"/ or romania/ or exp russia/ or rwanda/ or "saint kitts and nevis"/ or saint lucia/ or "saint vincent and the grenadines"/ or "sao tome and principe"/ or saudi arabia/ or serbia/ or sierra leone/ or senegal/ or seychelles/ or singapore/ or somalia/ or south africa/ or south sudan/ or sri lanka/ or sudan/ or suriname/ or syria/ or taiwan/ or tajikistan/ or tanzania/ or thailand/ or timor-leste/ or togo/ or tonga/ or "trinidad and tobago"/ or tunisia/ or turkmenistan/ or uganda/ or ukraine/ or united arab emirates/ or uruguay/ or uzbekistan/ or vanuatu/ or venezuela/ or vietnam/ or west indies/ or yemen/ or zambia/ or zimbabwe/ (1301281)

10 "organisation for economic co-operation and development"/ (557)

11 australasia/ or exp australia/ or austria/ or baltic states/ or belgium/ or exp canada/ or chile/ or colombia/ or costa rica/ or czech republic/ or exp denmark/ or estonia/ or europe/ or finland/ or exp france/ or exp germany/ or greece/ or hungary/ or iceland/ or ireland/ or israel/ or exp italy/ or exp japan/ or korea/ or latvia/ or lithuania/ or luxembourg/ or mexico/ or netherlands/ or new zealand/ or north america/ or exp norway/ or poland/ or portugal/ or exp "republic of korea"/ or "scandinavian and nordic countries"/ or slovakia/ or slovenia/ or spain/ or sweden/ or switzerland/ or turkey/ or exp united kingdom/ or exp united states/ (3501555)

12 european union/ (17739)

13 developed countries/ (21398)

14 or/10-13 (3517568)

15 9 not 14 (1211572)

16 8 not 15 (1472)

17 limit 16 to english language (1451)

NB: The search strategy for commentaries and narratives was date limited to retrieve references published 2020+ in order to retrieve the most relevant and current results. In addition, the validated NICE OECD countries geographic search filters by Ayiku, et al. (2021)^1^ were applied to the MEDLINE and Embase search strategies to retrieve results in the context of the UK and similar countries.

^1^Ayiku L, Hudson T, Williams C, Levay P, Jacob C. The NICE OECD countries' geographic search filters: Part 2-validation of the MEDLINE and Embase (Ovid) filters. J Med Libr Assoc. 2021 Oct 1;109(4):583-589. doi: 10.5195/jmla.2021.1224. <https://jmla.pitt.edu/ojs/jmla/article/view/1224>
