# Supplementary material for: Toward a New Conceptual Framework for Digital Mental Health Technologies: Scoping Review
Source: JMIR Ment Health. 2025 Feb 19;12:e63484. doi: 10.2196/63484 (PMC11864090; doi:10.2196/63484)
Supplement: Multimedia Appendix 2 [file mental-v12-e63484-s002.docx]

| **Domain identified within literature review** | **Number of breakout rooms that retained domains** | **Detail of breakout room notes** |
| --- | --- | --- |
| Condition | 4 out of 4 | Room 2, 3 and 4 – suggested the domain be expanded to include additional information on general characteristics (e.g. children and young people, vulnerable people), condition, and standalone symptoms |
| Setting | 2 out of 4 | Room 2 -suggested timing of use be included within condition, could be captured by setting |
| Platform / System / Technology | 4 out of 4 | Room 3 – suggested focus is on software unless specialist hardware is needed, suggested characteristics relating to AI should be captured |
| Function | 3 out of 4 | Room 3 – noted triage was an important function to include |
| Sub-function | 0 out of 4 | Rooms 2 and 3 – suggested function could capture key details |
| Professional input | 3 out of 4 | Room 2 – suggested domain renamed human input to reflect wider range of roles (e.g. peers)  Room 3 – suggested domain include information non referral routes  Room 4 – suggested domain renamed provider input and include information on type of professional and training |
| Type of communication | 1 out of 4 | Room 3 – suggested this could be captured in professional input |
| Type of intervention | 2 out of 4 | Room 3 – suggested that this could be captured in function but may also benefit from retaining to provide detail  Room 4 – suggested this could be captured by function |
| Sophistication | 1 out of 4 | Room 2 – suggested this is renamed to customisation and interactivity and could capture attributes relating to AI |
| Consumable Resource User | 1 out of 4 | Room 2 – suggested including professional time and other consumables, like hardware  Room 3 – suggested this could be captured in professional input |
